# Postoperative complications and antibiotic use in dogs with pyometra: a retrospective review of 140 cases (2019)

**DOI:** 10.1186/s13028-023-00670-5

**Published:** 2023-03-06

**Authors:** Outi Marita Turkki, Kristina Westberg Sunesson, Erik den Hertog, Katarina Varjonen

**Affiliations:** 1 AniCura Small Animal Hospital Bagarmossen, Ljusnevägen 17, 12848 Bagarmossen, Sweden; 2 Hertog Veterinary Research Support, Achterweteringseweg 30, 3738 MA Maartensdijk, the Netherlands; 3 AniCura Small Animal Hospital Albano, Rinkebyvägen 21B, 18236 Danderyd, Sweden

**Keywords:** Antibiotic guidelines, Complications, Canine, Peritonitis, Pyometra, Surgical site infection, SSI

## Abstract

**Background:**

Pyometra is commonly seen in intact bitches and is usually treated by ovariohysterectomy. Few studies have reported the frequency of postoperative complications, particularly beyond the immediate postoperative period. Swedish national antibiotic prescription guideline provides suggestions about which antibiotics should be used and when in individuals undergoing surgery. Studies on how well clinicians adhere to these guidelines, and on the outcome for these patients, have not been evaluated for cases of canine pyometra. This retrospective study conducted at a private Swedish companion animal hospital assessed complications that developed within 30 days of pyometra surgery, and whether clinicians followed the current national guidelines in regard to antibiotic use. We also assessed whether antibiotic use affected the rate of postoperative complications seen in this cohort of dogs, where antibiotics were predominantly used in cases presenting with a more severely depressed general demeanour.

**Results:**

The final analysis included 140 cases, 27 of which developed complications. In total, 50 dogs were treated with antibiotics before or during surgery and in 90 cases, antibiotics were either not given at all or treatment was initiated postoperatively (9/90) due to a perceived risk of infection developing. Superficial surgical site infection was the most common complication, followed by an adverse reaction to the suture material. Three dogs died or were euthanised during the immediate postoperative period. Clinicians adhered to national antibiotic prescription guidelines on when antibiotics should be given in 90% of cases. SSI only developed in dogs that were not given pre- or intra-operative antibiotics, while suture reactions did not appear to be affected by antibiotic use. Ampicillin/ amoxicillin was used in 44/50 cases given antibiotics before or during surgery, including most cases showing signs of concurrent peritonitis.

**Conclusion:**

Serious complications following the surgical treatment of pyometra were uncommon. Excellent adherence to national prescription guidelines was observed (90% of cases). SSI was relatively common and only seen in dogs that were not given antibiotics before or during surgery (10/90). Ampicillin/ amoxicillin was an effective first choice antimicrobial in cases requiring antibiotic treatment. Further studies are needed to identify cases benefiting from antibiotic treatment, as well as the duration of treatment needed to reduce the infection rate while also avoiding unnecessary preventive treatment.

**Supplementary Information:**

The online version contains supplementary material available at 10.1186/s13028-023-00670-5.

## Background

Pyometra is a common bacterial uterine infection reported to affect 20–25% of intact female dogs by 10 years of age [1–3]. When left untreated, the condition is fatal. Surgical ovariohysterectomy is currently considered the treatment of choice [2–5]. Multiple complications associated with pyometra have been reported, including peritonitis and cervical stump abscessation, wound infection, wound swelling, fistulous tract development, sepsis, haemorrhage, uveitis, conjunctivitis, pyelonephritis, arrhythmia, urinary tract infection and myocarditis [3, 6–8]. The prognosis following surgery is considered to be good. To the best of our knowledge, the frequency of postoperative complications arising within a 30-day period has not previously been reported.

It is currently open to debate whether all cases of pyometra should be treated with antibiotics in addition to surgical treatment. Some authors recommend that antibiotics are given in all cases [5, 9], whilst others recommend that antibiotics are only given to clinically unstable bitches with a risk of developing septicaemia [3, 10, 11]. The current literature recommends broad-spectrum antibiotics including second-generation cephalosporins, amoxicillin-clavulanic acid, metronidazole and enrofloxacin [9, 12]. A retrospective study published in 2014 reported the use of antibiotics in more than 50% of cases, either pre- or postoperatively [13].

Increasing concerns about antimicrobial resistance have prompted the publication of national and international guidelines for antimicrobial use. These aim to support clinicians in deciding when antibiotics should be prescribed and which antimicrobial should be the primary choice for various conditions [5, 14–17]. The most recent update to the Swedish antibiotic treatment guidelines, published in 2016, recommends perioperative antibiotics are only used in pyometra cases presenting with moderately to more severely depressed general demeanour. When treatment is required, ampicillin is considered to be the first-choice antimicrobial, and it should generally be discontinued postoperatively in cases where there is no critical illness with severe sepsis [14]. Due to a lack of published studies, these guidelines primarily rely on expert opinion. To date, no studies have assessed whether clinicians adhere to the guidelines in practice or evaluated the clinical outcome in cases where the guidelines have been followed.

This retrospective study explored how frequently complications including infection, haemorrhage, suture reaction, wound dehiscence and death were observed within 30 days following surgical treatment of pyometra in dogs in a Swedish companion animal hospital in 2019. In addition, we assessed whether clinicians at the hospital adhered to the current Swedish national antibiotic prescription guidelines when selecting antibiotic treatment and, if antibiotics were prescribed, which antibiotic was given, as well as the duration of treatment. We also assessed whether the frequency of postoperative complications differed between dogs prescribed pre- or intra-operative antibiotics and dogs not given antibiotics during this period. Using this information, we aimed to assess whether the current antibiotic guidelines, interpreted and applied by multiple clinicians, were sufficient for reducing the risk of inappropriate antibiotic use and preventing risk of infectious complications in this patient group.

## Methods

### Data collection

We retrospectively reviewed the medical records of canine cases diagnosed with pyometra and surgically treated with open abdominal ovariohysterectomy in 2019 in a Swedish small animal hospital. Cases for which a 30-day follow-up could not be obtained were excluded from further analysis.

The following descriptive information was collected from the digital medical records: breed, age, body weight, general demeanour, ASA (American Society of Anesthesiologists) classification, presence of peritonitis at the time of surgery, co-morbidities, pre-operative rectal temperature, visible vaginal discharge and concurrent conditions.

Postoperative complications recorded in the clinical file, including signs of surgical site infection (SSI), changes affecting the surgical site and other problems arising within a 30-day period following surgery were extracted. In cases where this information could not be obtained from the clinical records to cover a period of 30 days post-surgery, the information was obtained from the owners using an email questionnaire or via telephone (Additional file 1).

We obtained data relating to whether an antibiotic had been prescribed, the type of antibiotic treatment given, the time when antibiotic treatment was initiated in relation to the time of surgery, and the dosage and duration of antibiotic treatment given. Surgical details including information about the suture material and surgical method used, duration of the surgery and duration of follow-up were also collected for all cases.

### Allocation of patients into two groups

Patients were allocated into two groups based on antibiotic treatment before or during surgery. Patients not treated with antibiotics were placed in Group A whilst patients that had received antibiotics, initiated before or during surgery, were placed in Group B. Some of the cases in Group B were only given a single dose of antibiotics, whilst in other cases, treatment extended beyond the time of surgery. Treatment initiation was considered to be before or during surgery if the antibiotics were administered either within 24 h before the surgery or during surgery up until closure of the surgical site. Patients undergoing long-term antibiotic treatment for an unrelated disease were included in Group B but excluded from calculations about median and mean antibiotic treatment duration. Cases where antibiotic treatment was initiated after the closure of the surgical site were allocated to Group A and further assessed as cases with suspected infectious complications, as defined below.

### Assessment of adherence to national guidelines

The current Swedish antibiotic treatment guidelines for pyometra cases treated with surgery recommend that perioperative antibiotics are only used in cases presenting with moderately to more severely depressed general demeanour prior to surgery. We assessed adherence to the prescription guidelines based on the information in the clinical records. The assessment of the patient’s general demeanour prior to surgery in the clinical record was noted and compared to whether antibiotics had been prescribed to be given before or during surgery, or not initiated within this period. Cases with good general demeanour or mildly depressed demeanour that had been allocated to Group A based on antibiotic treatment as described above were considered to adhere to the guidelines, while cases allocated to Group B recorded as nondepressed or mildly depressed were considered not to adhere to the guidelines. Similarly, cases allocated to Group B with good or mildly depressed general demeanour upon clinical examination prior to surgery and cases allocated to Group A with moderately or more severely depressed general demeanour were considered not to adhere to the national guidelines.

### Definition of complications

A diagnosis of a postoperative surgical site infection (SSI) was based on guidelines from the Centers for Disease Control and Prevention (CDC) and adapted as stated below. Cases of postoperative surgical site infections were subdivided and defined as superficial, deep, or organ/space infections based on depth of infection. Infectious cases were categorised as belonging to one of these based on the clinicians’ descriptions in the dogs’ medical records. An infectious case was categorised as the deepest type of infection recorded, i.e., should a case show signs of both superficial and deep infection, it was categorised as a deep infection; a case of deep infection also showing signs of organ/space infection was considered an organ/space infection.

A superficial SSI was recorded if signs developed within 30 days of surgery, if only skin or subcutaneous tissue associated with the incision area were involved, and if at least one of the following was present: purulent discharge, a positive microbial culture from an aseptically obtained specimen from the wound, or wound drainage with pain, swelling, erythema or heat.

A deep SSI was recorded if it arose within 30 days of surgery and involved deep soft tissues and was associated with at least one of the following: purulent drainage from the deep incision, spontaneous wound dehiscence or an incision was opened and evidence of microbial infection detected, and pyrexia or tenderness was noted or an abscess or other evidence of deep infection was present.

An organ/ space SSI was recorded if the infection was observed within 30 days of surgery and involved part of the body deeper than fascia or muscle layers that had been affected during surgery and with at least one of the following: purulent drainage from organ/ space, positive microbiological sampling, or abscess or other evidence of infection, for example evidence on diagnostic imaging.

Evidence of increased haemorrhage developing following surgery, either subcutaneously or intra-abdominally, was recorded as a bleeding complication. Abnormal changes to the surgical site during the healing period were recorded as suture reactions when no signs of an SSI had been recorded or treated and the clinician had suspected a suture reaction.

Postoperative antibiotic treatment was defined as antibiotics initiated at any time following closure of the surgical site. Based on the group allocation described above, dogs prescribed postoperative antibiotics initiated after wound closure belonged to Group A and information about the cause of treatment initiation following surgery, as justified by the attending clinician in the clinical records, was extracted. In some cases, antibiotics had been prescribed during the postoperative period due to a perceived risk of or concern about infection, even when an infection had not been recorded or diagnosed. These cases were categorised as suspected complications as it was unclear whether a complication had developed. Dogs that died or were euthanised were recorded and, where available, the cause was noted.

### Statistical methods

Statistical analysis was performed using R (R version 4.0.4. and RStudio 1.4.1106). Baseline characteristics were expressed as n and % for count variables, as median and range for ordinal variables and for numeric variables with non-normal distribution, and as mean ± standard deviation for numeric variables with normal distribution. Normality was assumed when the histogram showed symmetry and the Shapiro-Wilk test was not significant. Baseline differences between dogs treated with antibiotics and dogs not treated with antibiotics were tested using the Wilcoxon Rank Sum test for ordinal and non-normally distributed numeric data, Fisher’s exact test for dichotomous and categorial data, and a two-sample t-test for numeric data that showed normal distribution. Welch’s correction was applied in cases of unequal variances in the treatment groups. P was set to < 0.05 for all analyses.

The primary outcome (complications: yes/no) was a composite outcome of all complications recorded, including surgical site infection, suture reaction, starting antibiotic treatment after surgery, death or euthanasia within 30 days following surgery. The effect of treatment was estimated using multiple logistic regression analysis (MRA) with complications (yes/no) as a dependent variable and the allocated treatment (pre/intraoperative antibiotics: yes/no) and possible confounders as independent variables. Confounding by indication could account for baseline imbalances. Only baseline characteristics that were considered relevant and could be measured were considered as possible confounders. This was based on the authors’ clinical experience as well as the availability of information in the clinical records. A variable was considered to be a confounder when: (1) it showed imbalance at baseline; (2) it had an association with the outcome and the allocated treatment; (3) there was a biologically plausible causal pathway from this variable to the outcome and to the allocated treatment.

## Results

### Study population

A search of the records identified 146 dogs diagnosed with pyometra and treated with ovariohysterectomy. Six dogs were excluded due to incomplete follow-up of complication risk calculations as the owners could not be reached to obtain 30-day post-surgical data.

The 140 remaining cases were available for a statistical analysis of complications. Of these 140 dogs, 29 were mixed breed and 111 were pure-breed dogs of 54 different breeds. Baseline characteristics of the study population and antibiotic groups are presented in Table 1. Group A consisted of 90 dogs and Group B of 50 dogs. Follow-up data covering 30 days post-surgery were available in the clinical records for 47/137 cases (excluding the three dogs that died or were euthanised within this timeframe). The authors contacted 78 owners by telephone, and 12 owners replied to an email questionnaire. The owners were contacted between 5 and 83 weeks after surgery (median 32 weeks).

Three dogs (2.1%) died or were euthanised without a conclusive diagnosis following surgery. These dogs all belonged to Group A. Two of these dogs originally presented with mild clinical signs and had not given rise to any particular concerns prior to or during surgery, whilst the third dog was recorded as presenting with severely depressed general demeanour and pyrexia. Of the two dogs with mild clinical signs, one was prescribed antibiotics postoperatively due to diarrhoea and was discharged from the hospital but subsequently died at home. One elderly dog failed to thrive within a few days of surgery, prompting the owners to request euthanasia. The third dog died in hospital on the following day after surgery.

Peritonitis was suspected in 17 cases during surgery and was later confirmed based on either peritoneal fluid cytology and/ or bacterial culture of peritoneal fluid. One of these dogs presented with good or mildly depressed general demeanour, 13 were considered moderately depressed and three were considered severely depressed. Antibiotic treatment was initiated in 16 of these dogs preoperatively or during surgery, and one dog started antibiotic treatment one day after surgery.

Statistically significant baseline differences were observed for ASA classification, preoperative rectal temperature, general demeanour and peritonitis present at the time of surgery. Dogs in Group B were more frequently classified as ASA $\ge$ 3 (OR= 4.69, 95% C.I: 1.71–13.69, P = 0.001), showed a slightly higher rectal temperature (mean 39.0 °C vs. 38.7 °C, P = 0.024), were more commonly depressed (moderate 70.0% vs. 5.6%, severe 16.0% vs. 2.2%) and were more frequently diagnosed with peritonitis at the time of surgery (32.0% vs. 1.1%, P < 0.001) compared to dogs in Group A.

**Table 1:** Baseline characteristics of 140 dogs treated surgically for pyometra, stratified by allocated treatment (pre/intraoperative antibiotics: yes/no)

	Group A (n = 90)		Group B (n = 50)		P-value
Age (in years; median, range)	9	1; 14	9	3; 15	0.433
Weight (in kg; median, range)	14	2; 65	10.5	2; 40	0.280
Type of pyometra (n, %)					0.111
Open	70	78.0	33	66.0	
Closed	19	21.0	17	34.0	
Not recorded	1	1.0	0	0.0	
ASA classification (n, %)					0.001*
< 3	43	47.8	10	20.0	
≥ 3	18	20.0	20	40	
Not recorded	29	32.2	20	40.0	
Preoperative temperature (in ℃; mean, sd)	38.7	0.70	39.0	0.81	0.019*
General demeanour (n, %)					< 0.001*
Good or mildly depressed	83	92.2	7	14.0	
Moderately depressed	5	5.6	35	70.0	
Severely depressed	2	2.2	8	16.0	
Concurrent diseases present (n, %)	41	45.6	28	56.0	0.290
Mammary tumour	11	12.2	6	12.0	1
Cardiac disease	11	12.2	6	12.0	1
Dermatological disease	8	8.9	2	4.0	0.495
Osteoarthritis	5	5.6	5	10.0	0.329
Other	16	17.8	14	28.0	0.198
Peritonitis present (n, %)	1	1.1	16	32.0	< 0.001*
Duration of surgery (in minutes; median, range)	60	10; 185	67.5	20; 235	0.055

Differences were statistically tested using Fisher’s exact test, the Wilcoxon Rank Sum test or Welch’s two-sample t-test

*Statistically significant at P < 0.05 level

### Surgery

The surgical preparation room, theatre room, surgical technique and choice of suture material, and drugs used for anaesthesia and pain relief all followed the hospital’s standardised procedure (Additional file 2). All dogs were treated with open abdominal ovariohysterectomy and ovarian pedicles were ligated with polydioxanone (PDS*II, Ethicon^®^) and abdominal walls were sutured with polydioxanone (PDS*II, Ethicon^®^) and poliglecaprone (Monocryl™, Ethicon^®^). The median duration of surgery was 60 min for Group A and 68 min for Group B (Table 1). A total of 26 clinicians were involved in the surgical management of cases.

### Antibiotic treatment and adherence to Swedish national antibiotic treatment guidelines

The Swedish antibiotic treatment guidelines regarding when antibiotic treatment should be initiated before or during surgery for cases of pyometra was followed in 90.4% of cases based on the clinicians’ recorded assessments of general demeanour prior to surgery.

Cases presenting with no or mild affectation to general demeanour were not prescribed antibiotics in 86/93 cases, whilst 46/53 cases showing moderate to severe affectation to clinical demeanour were prescribed antibiotics prior to surgery or during surgery based on clinical records. In 5/7 of cases with moderate or severe general demeanour affectation recorded, antibiotics were, based on the records, initiated following surgery due to post-surgical confirmation of bacteria on abdominal cytology sampled during surgery (n = 1), persistent high acute phase protein levels postoperatively (n = 1), antibiotic prophylaxis due to a subcutaneous hematoma (n = 1), urinary stones (n = 1) and postoperative diarrhoea (n = 1) followed by death. The dog with high acute-phase protein levels developed a suture reaction. Two of the cases were not given postoperative antibiotics, with no complications recorded.

Preoperative antibiotic treatment was initiated in 30/50 cases within 24 h of surgery, most commonly during the induction of anaesthesia, but in some cases the day before surgery. One dog was already on long-term antibiotic treatment for an unrelated disease before the diagnosis of pyometra. In 19/50 cases, antibiotic treatment was initiated during surgery due to suspected peritonitis (17/50) or a long duration of surgery and due to concerns that asepsis had been compromised (2/50). In 17/50 dogs, antibiotic treatment was not continued beyond the doses given before or during the surgery.

Ampicillin/ amoxicillin was used in 44/50 cases (median 19.5 mg/kg [range 8–25 mg/kg] three times daily) and marbofloxacin was used in one case. Treatment with ampicillin or amoxicillin combined with enrofloxacin (6 mg/kg once daily) or marbofloxacin (2.2 mg/kg once daily) was used in 4/50 cases. One dog already on long-term antibiotic treatment with metronidazole continued to receive this treatment. Thirty-two dogs (32/50), including cases presenting with peritonitis and excluding the dog on long-term antibiotic treatment were given antibiotics for a period of between 2 and 13 days with a median duration of 7 days. Of these dogs prescribed antibiotic treatment extending beyond the time of surgery, the reason for the prescription was clear in 30 cases and included moderately or severely depressed general demeanour, concurrent disease, peritonitis, contamination during surgery and combinations thereof. In two cases, the reason for antibiotic treatment could not be determined from the clinical records. Antibiotic treatment data for Group B are presented in Table 2, and details of antibiotic treatments are given in Additional file 3.

Septic peritonitis was suspected by the surgeon in 17 cases during surgery based on one or several macroscopic abdominal findings including inflammation of the parietal or visceral peritoneum, leakage of uterine contents from the oviduct or uterus, and presence of suspect exudate in the abdominal cavity. These cases were sampled and abdominal infection was confirmed postoperatively by cytology in one case and by positive culture in 16 cases. In 16 cases, antibiotic treatment was initiated prior to or during surgery (Group B), while in one case, it was initiated 1 day after (Group A). Twelve dogs were treated with ampicillin/ amoxicillin and four dogs were treated with a combination of ampicillin/ amoxicillin and enrofloxacin/ marbofloxacin. According to the clinicians’ recorded assessments, these four dogs showed severe signs of general infection. Antibiotics were given to all 16 dogs in Group B with peritonitis for 5–13 days, with a median of 7 days. The dog in Group A received ampicillin starting 1 day after surgery and the treatment continued for 2 days. The following bacteria were cultured: *Escherichia coli* (n = 9), *Streptococcus* spp. (n = 4), mixed bacteria (n = 2) and *Pasteurella* spp. (n = 1). A sensitivity analysis showed bacterial sensitivity to ampicillin/ amoxicillin for all cultured strains.

**Table 2:** Antibiotic treatment data for Group B (n = 50)

Antibiotic administration	Number of cases	Duration of treatment		Postoperative antibiotics > 24 h	
		< 24 h	> 24 h	Antibiotic	Duration (days) Median [range]
Time point for administration					
Preoperatively	30	16	14	Ampicillin/enrofloxacin	6.5 [2–12]
During celiotomy	19	1	18	Ampicillin/enrofloxacin	7 [5–13]
Ongoing long-term management	1	–	1	Metronidazole	> 30 days
Beta lactam					
Ampicillin iv. preoperatively	28	15	13	Amoxicillin	6 [2–12]
Ampicillin iv. during celiotomy	16	1	15	Amoxicillin	7 [5–13]
Fluoroquinolone ± beta lactam					
Ampicillin and enrofloxacin iv. preoperatively	1	–	1	Enrofloxacin	7 [7]
Ampicillin and enrofloxacin iv. during surgery	1	–	1	Enrofloxacin	5 [5]
Ampicillin and marbofloxacin iv. during surgery	2	–	2	Amoxicillin	9.5 [9, 10]
Marbofloxacin iv. preoperatively	1	1	–	–	–
Nitroimidazole					
Metronidazole ongoing long-term management	1	–	1	Metronidazole	> 30 days

Cases are expressed as numbers unless stated otherwise

### Complications

Postoperative complications were noted in 27/140 cases, and more than one complication was recorded in some cases. The observed complications are summarised in Table 3.

There was a statistically significant difference in the total number of dogs experiencing complications: three in Group B versus 24 in Group A (P = 0.003, Fisher’s exact test). In Group B, the only complications observed were suture reactions. Suture reactions were seen in seven cases, three in Group B and four in Group A, and these did not require treatment. Ten cases (all in Group A) developed a postoperative infection. Seven of these ten dogs developed a superficial SSI 4–7 days following surgery, four of them required repeat anaesthetic for wound treatment, drainage or repair due to superficial wound dehiscence, and three cases required medical treatment in combination with local wound treatment for second-intention healing. Three dogs developed an organ/ space infection: in one case, a uterine stump infection was recorded, the second case presented with a uterine stump infection and peritonitis, and wound dehiscence and abdominal herniation with peritonitis were diagnosed in the third case. In 71/137 cases, the dogs did not receive antibiotics and did not show signs of SSI. No cases showed clinical signs of postoperative abdominal haemorrhage. One dog developed subcutaneous bleeding but no signs of SSI.

Nine elderly dogs (median age 11 years, range 9–13) were prescribed postoperative preventive antibiotics initiated between 1 and 3 days following surgery. These dogs were classified into Group A due to the delayed start of antibiotic treatment. They were categorised as suspected complications and antibiotic treatment was initiated following surgery due to a perceived risk of infection developing. Recorded reasons for initiating treatment included abdominal discomfort on palpation following surgery (n = 1), persistently high acute-phase protein levels postoperatively (n = 2), antibiotic prophylaxis due to a subcutaneous haematoma (n = 1), urinary stones (n = 1), liver pathology (n = 1) and liver pathology combined with bacteriological findings from abdominal cytology during surgery (n = 1), while in one case, no reason was recorded (n = 1). One dog was started on antibiotics due to postoperative diarrhoea and died (as described above). There were no further complications observed in the remaining eight dogs.

**Table 3:** Reported complications in 140 dogs treated surgically for pyometra, stratified by allocated treatment (pre/intraoperative antibiotics: yes/no)

	Group A (n = 90)		Group B (n = 50)		P-value
Complication (n, %)	24	26.7	3	6.0	0.003*
SSI (n, %)	10	11.1	0	0.0	0.014*
Superficial SSI	7	7.8	0	0.0	0.049*
Deep SSI	0	0.0	0	0.0	NA
Organ/ space SSI	3	3.3	0	0.0	0.300
Death or euthanasia (n, %)	3	3.3	0	0.0	0.553
Starting postoperative antibiotic (n, %)	9	10.0	0	0.0	0.025*
Suture reaction (n, %)	4	4.4	3	6.0	0.706

One dog could have more than one complication. Differences between the treatment groups were statistically tested using Fisher’s exact test

*Statistically significant at P < 0.05 level

### Comparison of complication rates between Group A and Group B

Dogs assigned to pre- or intraoperative antibiotic treatment based on the clinicians’ judgement had 5.6 times lower odds for developing one or more postoperative complications compared to dogs in Group A (unadjusted OR 0.18, 95% C.I.: 0.04; 0.54, P = 0.007).

When adjusted for ASA classification, preoperative body temperature, general demeanour and presence of peritonitis, this decrease in odds was even more pronounced (adjusted OR 0.06, 95% C.I.: 0.00; 0.48, P = 0.019).

The difference in the number of SSIs between Group A (10/90) and Group B (0/50) can be estimated by Fisher’s exact test as an odds ratio of 0.00 (95% CI: 0.00;0.73, P = 0.014). We used logistic regression in order to be able to include multiple variables. We planned to estimate the odds ratio using logistic regression but were unable to do so because no SSI were observed in Group B.Therefore, we did not adjust for confounding baseline differences for the odds ratio for SSI.

### Sensitivity analysis

We performed a non-*a-priori* planned sensitivity analysis to investigate whether the results of the comparison in complication rate between Group A and Group B were robust to the exclusion of the nine elderly dogs for whom antibiotics were prescribed during the early postoperative period without apparent signs of SSI. When these nine dogs were excluded from the analysis, the difference in the number of SSI between Group A (10/81) and Group B (0/50), as estimated by Fisher’s exact test, was an odds ratio of 0.00 (95% CI: 0.00; 0.65, P = 0.007). Furthermore, when the nine dogs were excluded, the differences in total complication rates between the treatment groups, as estimated by logistic regression analysis, became insignificant (unadjusted OR 0.28, 95% C.I.: 0.06; 0.91, P = 0.055; adjusted OR 0.97, 95% C.I.: 0.05; 16.31, P = 0.982).

## Discussion

A complication developed within 30 days of surgery in 27/140 cases (19.3%) in this cohort. SSI was the single most common complication, seen in 7.1% of cases, followed by suture reactions in 5.0% of cases. The latter resolved without further treatment, whilst infections were treated with either a combination of repeat surgery and antibiotic treatment or antibiotic treatment alone.

The overall complication rate for surgically treated pyometra is reported to be up to 25% [6, 11]. In our study, we did not include conditions observed at the time of surgery (such as peritonitis diagnosed pre- or during surgery) as a complication, but focused on complications seen following surgery and developing over the following 30-day period, thus making it difficult to compare these studies [6]. The mortality rate in our study was 2.1%, which is similar to previously reported figures showing mortality rates below 4.5% [1, 6, 7].

The study material revealed statistical differences between cases in the group given antibiotics (Group B) and the group not given antibiotics before or during surgery (Group A). We divided our cases into two treatment groups based on whether or not the clinicians decided to prescribe antibiotics before/ during surgery. Cases with a more depressed demeanour, given a higher ASA classification, with a higher body temperature and showing evidence of peritonitis were more likely to belong to Group B. Starting antibiotics before surgery or giving antibiotics during the surgical procedure to patients with a more depressed general demeanour is in accordance with current treatment guidelines. This study can therefore not be seen as a comparison of outcomes between comparable severely diseased cases receiving or not receiving antibiotics, and groups must be studied separately in terms of a risk of complications.

The need to use antibiotics as a routine preventive treatment during different types of surgery has been challenged in recent years, with the aim to reduce antibiotic use, particularly in sterile surgeries [18–25]. As a result, fewer cases are routinely given antibiotics, and shorter treatment durations are prescribed. To our knowledge, no such studies have yet been published for cases of pyometra. Pyometra surgery is not considered a sterile surgery due to the exposure of the cervical contents to the abdominal cavity when the uterus is removed, and the risk of sepsis and toxaemia [13, 26]. Severe uterine endometrial inflammation with bacterial growth and accumulation of pus can frequently lead to endotoxaemia and an immune response affecting the animal systemically [3, 5, 26]. Despite this, the need for routine antibiotic treatment is discussed in the literature, with some authors proposing that it is not always required, particularly in cases where there is no concern for sepsis and the dog is considered stable [3, 10].

Dogs presenting with good or mildly depressed general demeanour were not prescribed antibiotics in 86/93 cases, whilst 46/53 cases showing moderately to severely depressed general demeanour were prescribed antibiotics with a 90% adherence to the current Swedish antibiotic guidelines [14], suggesting a good acceptance of the current guidelines amongst clinicians. In addition to moderately or severely depressed general demeanour, other reported causes for antibiotic treatment before or during surgery included signs of peritonitis. Ampicillin/ amoxicillin was chosen in almost 90% of cases where antibiotics were used, in accordance with the guidelines. In a large study on antimicrobial surgical prophylaxis in humans [21], the antimicrobial agents prescribed were in accordance with the published guidelines for more than 90% of patients, a result very similar to what we found in our study.

All cases of SSI developed in dogs belonging to Group A. Postoperative SSIs were seen in 10/90 (11%) of cases not treated with antibiotics preoperatively or during surgery. This reflects a relatively high risk, particularly considering that no SSIs developed in Group B despite most of these dogs having been considered at risk of sepsis, and bacterial peritonitis was present in a third of the cases. A previous study reported wound infections in 3.0% of cases [6]. When including all the dogs that received antibiotics perioperatively, our results were similar. In our study, 5.0% of the dogs developed a superficial SSI, compared to the slightly lower rate of 3% in the previous study, and post-discharge deep organ/ space SSIs (peritonitis and cervical stump infections) were recorded in 2.1% (3/140) of our cases and in 0.5% (2/315) of cases recorded by Jitpean et al. [6].

The relatively high frequency of SSI seen in cases not given preventive antibiotic treatment in this study raises the question of why these infections develop in cases that the clinician in charge has assessed to be stable and with good general demeanour pre-surgery. One potential cause identified was a variation in when the decision of whether or not to administer antibiotic treatment was made. In some cases, the decision was made at presentation by the admitting clinician based on general demeanour on arrival. However, in other cases, the assessment had not been made until the dog had already received some pre-operative treatment such as intravenous fluids and pain relief, potentially improving the general demeanour of a dog with more severe disease that would, if an assessment and decision had been made upon arrival to the hospital, have prompted the use of antibiotics. Furthermore, 26 clinicians were involved in assessing the general demeanour of these animals. General demeanour is a subjective interpretation of the patient made within a short time frame and in some cases, signs of more severe depression may be masked during the examination due to the stress associated with transportation to the hospital and the examination itself. The decision about whether or not to administer pre- or intraoperative antibiotic treatment will therefore likely vary among clinicians based on their interpretation of clinical signs as well as the information provided by the owner. Individual patients might be interpreted as less severely affected due to variations in behaviour based on breed, age and temperament, as well as the dog’s ability to mask signs of more severe disease when faced with stress brought on by a hospital environment. Assessment is also based on the owner’s ability to describe the change they have noted and the clinician’s interpretation of this information. Additional criteria are therefore needed to help with the assessment.

Our study included 90 dogs that were not treated with antibiotics before or during surgery. Infectious complications were found to have developed in 11% of the cases in this group. All dogs with an infection were recorded to have good or mildly depressed general demeanour following surgery. An increased risk of infections developing (including at a later date) has been reported as a risk in patients with sepsis [27]. Sepsis is an organ dysfunction caused by dysregulated host responses to infection and will, in addition to an acute life-threatening disorder, cause profound immunosuppression observed long after recovery [27]. General demeanour alone is unlikely to be an adequate criterion for detecting all septic individuals. Although sepsis/ septic shock is fairly straightforward to detect clinically in severely ill patients with e.g. fever, altered mentation, tachycardia/ bradycardia, tachypnoea/ bradypnea, abnormal pulse quality, hypotension, pale or hyperaemic mucus membranes, high/ low white blood cell (WBC) count and hypoglycaemia, it may be more difficult to diagnose in cases where signs are more vague or where sepsis is still developing [28]. Hauptman et al. [29] defined the risk of sepsis in dogs as cases where abnormalities can be seen in two out of the following four parameters: rectal temperature, WBC count, heart rate and respiration rate. The same classification is also used in humans [30].

Biomedical markers have been studied to try and find unique markers to diagnose infection and detect the risk of sepsis. Acute-phase proteins, cytokines and chemokines have been studied [26, 31–35], while several studies also report a correlation with plasma C-reactive protein (CRP) and pyometra [13, 26, 33, 34]. However, two studies reported that CRP was not significantly different in septic and non-septic bitches with pyometra [13, 34]. Significant differences in serum amyloid A (SAA) have been indicated between septic and non-septic animals [13]. A recent study from Ahn et al. [31] showed correlations between CRP, SAA and cell-free DNA (cfDNA) levels and pyometra. Only cfDNA correlated with WBC counts, which is a criterion for sepsis. Although measurements of cfDNA, certain proteins and sepsis-specific cytokines may be clinically useful for prognosis, these tests are not currently available in clinical practice [31, 32, 34].

Ampicillin/ amoxicillin effectively prevented the development of SSI complications in all cases in Group B, including the 17 cases given a single dose of antibiotics at the time of surgery. The majority of these cases presented with moderately or severely depressed general demeanour. In Group B (n = 50), ampicillin/ amoxicillin was chosen preoperatively or during surgery in 88.0% of cases given antibiotics. Fluroquinolones were used in only 10.0% of Group B cases before or during surgery, which likely reflects the Swedish national legislation from 2013, which states that this group of antibiotics should only be prescribed for cases with life-threatening infectious disease, where there is good reason to believe other options would not work, or cases where culture and sensitivity tests indicate that other options are not viable, thus limiting the routine use of this group of antibiotics [36].

Interestingly, all 17 cases where septic peritonitis was suspected during surgery and later confirmed recovered after antibiotic treatment. Treatment recommendations for pyometra found in the literature suggest using a broad-spectrum antimicrobial with an antibacterial spectrum against gram-positive, gram-negative and anaerobic bacteria whilst waiting for the results of culture and sensitivity testing [14]. Amoxicillin-clavulanic acid, enrofloxacin and metronidazole have been suggested [12], as well as third-generation cephalosporins or ampicillin combined with an aminoglycoside [37]. In our study, antibiotic treatment was initiated before or during surgery in 16/17 cases of peritonitis and the day after surgery in one case. In 13/17 cases, ampicillin/ amoxicillin was used as the sole antibiotic treatment, and in four cases, treatment was initiated with a combination of ampicillin/ amoxicillin and enrofloxacin/ marbofloxacin, but continued only with ampicillin/ amoxicillin. In this cohort, ampicillin/ amoxicillin treatment (median 7 days, range 2–13) seemed sufficient for treatment of bacterial peritonitis. This observation is supported by a bacteriological study from 2005 [38], in which uterine infectious agents, mainly *E. coli*, showed low resistance to ampicillin (10%) and enrofloxacin (4%). In our study, the group of dogs showing signs of peritonitis was too small to draw firm conclusions, but our results indicate that ampicillin/ amoxicillin remains a good antimicrobial choice in cases presenting with peritonitis associated with pyometra in this region of the world.

Nine dogs in this cohort that received postoperative antibiotic treatment were prescribed antibiotics within 3 days of surgery based on the clinicians’ assessment of the risk of infection developing. An infection was not confirmed in any of these cases as they did not fulfil CDC criteria for SSI, but it is possible that at least some of these dogs either showed early signs of infection or would have developed an infection if not treated. It seems likely that some of these dogs did not have an infection, thus reducing the risk of complications following surgery, but this remains speculative.

The main limitation to our study is its retrospective nature, relying on information that could be extracted from clinical records. Based on the results of this study alone, it is not possible to determine whether initiating antibiotic treatment before or during surgery alone, without continuing the treatment, would be sufficient in preventing postoperative infectious complications in cases where antibiotics are required. A future prospective study assessing the duration of antibiotic treatment needed for this patient group should therefore be performed. If perioperative treatment alone proves sufficient in preventing infections, it is possible that a reduction in the overall use of antibiotics could be achieved by shortening the duration of treatments given and by reducing the need for antibiotic use in cases that develop SSI. Additionally, identifying useful biochemical parameters and making these available for use in practice could further support clinical findings (e.g. general demeanour) in order to detect patients with more vague clinical signs of sepsis or those in the process of developing sepsis, thus helping to reduce the risk of infectious complications arising and improve guidelines.

This study supports previous findings that complications following pyometra surgery are rarely severe and that antibiotics are not always required. The relatively high infectious complication rate seen in cases considered stable prior to surgery and therefore not given antibiotics is cause for concern. Identification of additional criteria to help predict outcome and support clinicians when assessing which cases might be at risk of developing SSI would therefore be valuable to improve national antibiotic guidelines. Prospective studies assessing the appropriate duration of antibiotic treatment in cases requiring antibiotics should also be conducted.

## Conclusion

SSI was the most common complication noted, in this material only seen in cases not given preventive antibiotic treatment. However, half of the cases recovered well without antibiotic treatment, emphasising the fact that antibiotics are not needed in every case. This study highlights the need to improve the process of identifying cases that would benefit from antibiotic treatment in order to reduce risk of infections developing in the surgical area following pyometra surgery. Identification of reliable biomarkers and the availability of such markers to identify patients at risk of sepsis in practice could help clinicians reduce the risk of postoperative infectious complications. Ampicillin/ amoxicillin remains the preventive treatment of choice in cases considered to be at risk of postoperative infection developing based on pre-operative status, and in cases with overt peritonitis observed at the time of surgery in this region of the world.

## Supplementary information

**Additional file 1**: Questionnaire for telephone interview or email contact (translated from Swedish).

**Additional file 2**: Surgical approach. 

**Additional file 3**: Antibiotic treatment.

## Data Availability

Detailed data used in this study can be requested from the corresponding author.

## Abbreviations

ASA: American Society of Anesthesiologists
CDC: Centers for Disease Control and Prevention
cfDNA: Cell-free DNA
CRP: C-reactive protein
iv.: Intravenously
MRA: Multiple logistic regression analysis
SAA: Serum amyloid A
SSI: Surgical site infection
WBC: White blood cell
