# Supplementary material for: Postoperative complications and antibiotic use in dogs with pyometra: a retrospective review of 140 cases (2019)
Source: Acta Vet Scand. 2023 Mar 6;65:11. doi: 10.1186/s13028-023-00670-5 (PMC9987112; doi:10.1186/s13028-023-00670-5)
Supplement: Supplementary file 1 — Additional file 1: Questionnaire for telephone interview or email contact (translated from Swedish). [file 13028_2023_670_MOESM1_ESM.docx]

**Additional file 1. Questionnaire for telephone interview or email contact (translated from Swedish)**

Has your dog experienced any issues with wound healing within the first 14 days post-surgery?

Answer:

If yes, can you please describe in your own words what the wound looked like?

After the initial 14-day post-surgery period, did your dog experience any issues with the wound?

Answer:

If yes, can you please describe in your own words what the wound looked like?

Approximately how long after surgery did you notice this change?

Did your dog develop an infection in the abdomen at any point following the surgery?

Answer:

If yes, please specify what type of infection (for example peritonitis, cervical stump infection)?

Approximately how long after the surgery did this occur?
