# Supplementary material for: Postoperative complications and antibiotic use in dogs with pyometra: a retrospective review of 140 cases (2019)
Source: Acta Vet Scand. 2023 Mar 6;65:11. doi: 10.1186/s13028-023-00670-5 (PMC9987112; doi:10.1186/s13028-023-00670-5)
Supplement: Supplementary file 2 — Additional file 2: Surgical approach. [file 13028_2023_670_MOESM2_ESM.docx]

**Additional file 2. Surgical approach**

Dogs were rehydrated with intravenous fluids (Ringeracetat, Fresenius Kabi) prior to anaesthesia. The anaesthesia was induced with propofol (PropoVet™ Multidose, Zoetis) or alfaxalone (Alfaxan Multidose, Orion Pharma Animal Health) intravenously. Methadone (Semfortan vet, Dechra Veterinary Products) was administered intramuscularly pre-surgery and complemented with fentanyl (Fentanyl B. Braun, B. Braun) during surgery where necessary. Following induction, isoflurane (Attane vet, VM Pharma) inhalation was used to maintain anaesthesia during surgical preparation, followed by sevoflurane (SevoFlo®, Zoetis) once the patient was transferred into theatre. Intravenous fluids were continued during surgery. The surgical site was clipped and prepared with chlorhexidine acetate soap (Klorhexidin Fresenius Kabi, Fresenius Kabi) and the skin incision site was anaesthetised using lidocaine administered subcutaneously (Xylocain®, Aspen Nordic). Chlorhexidine acetate cutaneous solution (Klorhexidin Fresenius Kabi, Fresenius Kabi) was applied to the surgical site and left to dry prior to draping the patient.

Open abdomen ovariohysterectomy was performed according to standard techniques. Polydioxanone (PDS*II, Ethicon®) was used to ligate the ovarian pedicles, broad ligaments and cervix.

The linea alba in the abdominal wall was sutured with polydioxanone (PDS*II, Ethicon®) with a continuous pattern in all cases, while subcutaneous tissue was sutured continuously with polydioxanone (PDS*II, Ethicon®) or poliglecaprone (Monocryl™, Ethicon®), and the dermis with an intracutaneous suture pattern with poliglecaprone (Monocryl™, Ethicon®). The wound was covered with a sterile patch (Mepilex, Mölnlycke®) for 48 hours in accordance with the hospital’s standard procedure.

The owners were instructed to monitor the dog’s body temperature over the following week, to check the wound daily, and to contact the hospital should any signs of discharge, wound site swelling or other cause of concern arise. An Elizabethan collar was recommended for 10–14 days and walks on a leash for 3 weeks. A re-examination appointment was recommended 10–14 days following surgery.
