# Supplementary material for: Postoperative complications and antibiotic use in dogs with pyometra: a retrospective review of 140 cases (2019)
Source: Acta Vet Scand. 2023 Mar 6;65:11. doi: 10.1186/s13028-023-00670-5 (PMC9987112; doi:10.1186/s13028-023-00670-5)
Supplement: Supplementary file 3 — Additional file 3: Antibiotic treatment. [file 13028_2023_670_MOESM3_ESM.docx]

**Additional file 3. Antibiotic treatment**

The intravenous beta-lactam treatment was initiated with ampicillin (Doctacillin®, Meda)

at a dose of 20–30 mg/kg every 90 minutes during the surgery. If intravenous treatment was continued, the same product and dose was administrated four times per day. Post-discharge treatment with oral amoxicillin (Vetrimoxin®, Cefa Animal Health) 20 mg/kg was administered three times per day.

Fluroquinolones were used at the following doses: enrofloxacin (Baytril®Vet, Elanco) 5 mg/kg iv., or marbofloxacin (Marbocyl®, Vetoquinol) 2.1–2.3 mg/kg iv. once daily with or without ampicillin (Doctacillin®, Meda) 20–30 mg/kg iv. every 90 minutes during the surgery and four times per day during hospitalisation. Post-discharge treatment was maintained at the following doses: enrofloxacin (Baytril®Vet, Elanco) 5 mg/kg or marbofloxacin (Marbocyl®, Vetoquinol) 2–5 mg/kg po. once daily or amoxicillin (Vetrimoxin®, Cefa Animal Health) 20 mg/kg three times per day.
